# Polymorphism of the *p38β* gene in patients with colorectal cancer

**DOI:** 10.3892/ol.2014.2315

**Published:** 2014-07-04

**Authors:** JAN DIMBERG, RENATE SLIND OLSEN, MARITA SKARSTEDT, STURE LÖFGREN, NIKLAS ZAR, ANDREAS MATUSSEK

**Affiliations:** 1 Department of Natural Science and Biomedicine, University College of Health Sciences, Jönköping, SE-551 11, Sweden; 2 Department of Laboratory Services, Ryhov County Hospital, Jönköping, SE-551 85, Sweden; 3 Division of Drug Research, Department of Medical and Health Sciences, Faculty of Health Sciences, Linköping University, Linköping SE-581 85, Sweden; 4 Department of Clinical Microbiology, Ryhov County Hospital, Jönköping, SE-551 85, Sweden; 5 Department of Surgery, Ryhov County Hospital, Jönköping, SE-551 85, Sweden

**Keywords:** *p38β*, promoter region, single nucleotide polymorphism, colorectal cancer

## Abstract

The p38 mitogen-activated protein kinase (MAPK) signaling pathways have been proposed to participate in the pathological process of cancer by affecting inflammation, proliferation, metastasis and cell survival. A single nucleotide polymorphism (SNP; *rs2235356*, −*1628A*→*G*) in the promoter region of the *p38β* gene has been proposed as a genetic modifier for colorectal cancer (CRC) in a Chinese population. The present study evaluated the susceptibility of patients possessing this SNP to CRC, in addition to determining its association with clinical parameters in Swedish patients with CRC. Using the LightSNiP genotyping assay, this SNP was screened in 389 patients with CRC and 517 control subjects. No significant difference in the genotype distribution or in the allelic frequencies was identified between the two groups nor was any association identified with the clinical parameters. These findings indicate that the −*1628A*→*G* polymorphism of the *p38β* gene is not significantly associated with a susceptibility to CRC in a Swedish population.

## Introduction

There are numerous genetic pathways, which affect colorectal cancer (CRC) initiation and progression. These include the chromosomal instability pathways, which are associated with accumulation of activating mutations in oncogenes, such as *KRAS*, *BRAF* and *PI3KCA* or inactivation of tumor suppressor genes, such as *APC* and *p53* (1,2). Furthermore, aberrant methylation of gene promoter regions has been widely investigated and these epigenetic events within certain signaling pathways result in gene suppression and contribute to the development of CRC (3,4).

Genetic variation, such as single nucleotide polymorphisms (SNPs), is hypothesized to contribute to individual variations in CRC susceptibility (5). Polymorphic variants of genes are significant factors that mediate inflammatory responses and facilitate CRC progression (6). The prognosis of CRC is dependent on the extent of local and metastatic tumor spread and the degradation of the extracellular matrix (ECM) that surrounds cancerous tissue. Matrix metalloproteinases (MMPs) have fundamental roles in the degradation of basal membranes and the ECM, as well as being associated with tumor invasion and a poor prognosis (7). Our previous study demonstrated that the gene polymorphism of the *MMP12* gene is associated with a higher risk of disseminated CRC (8).

Mitogen-activated protein kinase (MAPK) signaling pathways (2,9) consist of a large family of serine-threonine kinases, which respond to extracellular signals, such as cellular stress and growth factors. These signals control fundamental cellular processes, such as cell proliferation, differentiation, cell survival and are considered to be significant in the progression of different types of cancer (10), including CRC (2,9,10). p38 kinases are members of the MAPK family and consist of four isoforms (α, β, γ and δ) (9–11). An increased expression level of *p38* has been observed in CRC (12) and breast cancer (13) patients.

Recently, it was shown that an SNP, *rs2235356*, −*1628A*→*G*, in the promoter region of the *p38β* gene was correlated with an increased risk of CRC in a Chinese population (14). In the present study, this SNP was analyzed to assess its value as a risk factor and as a predictor of disease outcome in Swedish CRC patients.

## Patients and methods

### Patients, controls and ethical procedures

The present study collected blood samples from 389 consecutive patients from southeastern Sweden who underwent surgical resection for primary colorectal adenocarcinomas at the Department of Surgery, Ryhov County Hospital (Jönköping, Sweden) between 1996 and 2013. Clinicopathological characteristics of the patients were obtained from surgical and pathological records. The investigation was approved by the local Ethical Committee of the Faculty of Health Sciences (Dnr. 2013/271-31; Linköping, Sweden) and informed consent was obtained from the participants.

### Patient groups

The patients comprised 212 males and 177 females with a mean age of 70 years (range, 25–93 years). The tumors were located in the colon in 218 cases and in the rectum in 171 cases, and were classified by Dukes’ classification system (15): Stage A, n=65; stage B, n=144; stage C, n=121; and stage D, n=59. Blood donors (n=517) with no known history of CRC, who were from the same geographical region as the patients with CRC were selected as control subjects. The control group consisted of 255 males and 262 females, with a mean age of 60 years (range, 33–79 years). Blood samples were centrifuged using Hettich Universal 320R Centrifuge (Andreas Hettich GmbH & Co. KG, Tuttligen, Germany) at 1500 × g for 10 min to separate the plasma and blood cells and were stored at −78°C.

### DNA extraction and genotype determination

Genomic DNA was isolated from the blood samples using the QIAamp DNA Blood kit (Qiagen, Valencia, CA, USA). DNA samples were genotyped using the LightSNiP rs2235356 genotyping assay (TIB Molbiol, Berlin, Germany). DNA (10 ng) was mixed with Reagent Mix, LightCycler^®^ FastStart DNA Master HybProbe (Roche Diagnostics GmbH, Mannheim, Germany), 5.7 μl H_2_O and 0.8 μl MgCl_2_ and amplified using the LightCycler^®^ 480 Real-Time PCR system (Roche Diagnostics GmbH) according to the manufacturer’s instructions.

### Statistical analysis

Differences in the frequencies of the *p38β* gene polymorphism between patients and the control group, as well as between clinical characteristics within the CRC subgroup were analyzed using the χ^2^ test and the Hardy-Weinberg equilibrium was assessed with regard to the genotypes. Survival analysis was performed using Cox’s regression and Kaplan-Meier analysis was conducted with the log-rank test. Statistical analysis was performed using SPSS statistical software (version 19; IBM, Armonk, NY, USA) and P<0.05 was considered to indicate a statistically significant difference.

## Results

### Genotype distribution

The frequencies of the gene polymorphisms in the patients and the control group subjects indicated no significant difference in the genotype distribution or allelic frequencies (Table I). The genotypes in the CRC patients and the control group subjects were not associated with clinical characteristics, such as age and gender (data not shown). When subdividing the patients into groups of colon and rectal cancer, or localized Dukes’ A and B and disseminated Dukes’ C and D disease, no significant difference was identified between location and disease with regard to the genotypes and alleles (Table II). In addition, analysis using the Kaplan-Meier method demonstrated that the *p38β* genotypes were not associated with survival rate (data not shown).

Neither the patient nor the control group showed a significant deviation in genotypic frequency as assessed by the Hardy-Weinberg equilibrium (data not shown).

## Discussion

The p38 MAPKs are involved in inflammation, proliferation, differentiation, and cell death (2,9–11) and increased levels have been observed in CRC patients (12). MMPs are fundamental in the degradation of the ECM and are associated with tumor invasion and a poor prognosis (7). The p38 MAPK signaling pathways have been proposed to promote metastasis (16) by activating different transcription factors. One of these transcription factors, activator protein-1 (AP1) (11,16) appears to be important, as it controls MMP expression (16–19) and may impact CRC progression via tumor invasion (20).

To the best of our knowledge, studies are limited regarding the association between p38 genetic polymorphisms and the risk of CRC. However, it was recently demonstrated that an SNP (*rs2235356*, −*1628A*→*G*) in the promoter region of the *p38β* gene was associated with an increased risk of CRC in a Chinese population (14). To elucidate whether this SNP is correlated with the risk of CRC in other ethnic groups, this particular SNP was analyzed in Swedish patients with CRC. In the present study, clinicopathological variables, such as tumor localization, stage and survival were evaluated, which is similar to a previous study by Huang *et al* (14).

In the current study, it was identified that the genotype distribution and allelic frequencies of patients were not significantly associated with CRC when compared with the control subjects. Functionally, the −*1628A*→*G p38β* gene polymorphism may influence CRC progression via the p38 MAPK signaling pathways as a regulator of the proliferation, survival and metastasis. In addition, no significant correlation between the genotypes and survival rate or disseminated disease was detected. Furthermore, the genotypes were not identified to be associated with other clinical parameters.

In conclusion, the present study indicated that the −*1628A*→*G* polymorphism in the promoter region of the *p38β* gene does not appear to be a useful tumor marker that reflects the clinical outcome in Swedish patients with CRC. In the future it would be of interest to evaluate this SNP in a large cohort of various ethnicities.

## Figures and Tables

**Table I:** Genotypic and allelic distribution of the *p38β* gene polymorphism (−*1628A*→*G*) in patients with CRC and in healthy control subjects.

A, Genotype distribution		

A→G	CRC, % (n=389)	Controls, % (n=517)
A/A	28.3 (110)	24.9 (129)
A/G	45.5 (177)	51.3 (265)
G/G	26.2 (102)	23.8 (123)

B, Allele distribution		

Variant	CRC, % (n=778)	Controls, % (n=1034)

A	51.0 (397)	50.6 (523)
G	49.0 (381)	49.4 (511)

CRC patients vs. control subjects: Genotype and allele distribution, no significant differences (P>0.05). CRC, colorectal cancer.

**Table II:** Genotypic and allelic distributions of the *p38β* gene polymorphism (−*1628A*→*G*) regarding tumour location and disease stage in patients with CRC.

A, Genotype				

	Location		Stage	
		
A→G	Colon % (n)	Rectum % (n)	Dukes’ A + B % (n)	Dukes’ C + D % (n)
A/A	28.4 (62)	28.1 (48)	28.2 (59)	28.3 (51)
A/G	45.0 (98)	46.2 (79)	46.0 (96)	45.0 (81)
G/G	26.6 (58)	25.7 (44)	25.8 (54)	26.7 (48)
Total	(218)	(171)	(209)	(180)

B, Allele				

	Location		Stage	
		
Variant	Colon % (n)	Rectum % (n)	Dukes’ A + B % (n)	Dukes’ C + D % (n)

A	50.9 (222)	51.2 (175)	51.2 (214)	50.8 (183)
G	49.1 (214)	48.8 (167)	48.8 (204)	49.1 (177)
Total	(436)	(342)	(418)	(360)

Colon vs. rectum and Dukes’ A + B vs. Dukes’ C + B: Genotype and allele distribution, no significant differences (P>0.05). CRC, colorectal cancer.
